# Comprehensive Evaluation of *TFF3* Promoter Hypomethylation and Molecular Biomarker Potential for Prostate Cancer Diagnosis and Prognosis

**DOI:** 10.3390/ijms18092017

**Published:** 2017-09-20

**Authors:** Maibritt Nørgaard, Christa Haldrup, Tine Maj Storebjerg, Else Marie Vestergaard, Peter J. Wild, Søren Høyer, Michael Borre, Torben Falck Ørntoft, Karina Dalsgaard Sørensen

**Affiliations:** 1 Department of Molecular Medicine, Aarhus University Hospital, 8200 Aarhus N, Denmark; mnj@clin.au.dk (M.N.); christa.haldrup@clin.au.dk (C.H.); tinestor@rm.dk (T.M.S.); Orntoft@clin.au.dk (T.F.Ø.); 2 Department of Histopathology, Aarhus University Hospital, 8200 Aarhus N, Denmark; soren.hoyer@gmail.com; 3 Department of Urology, Aarhus University Hospital, 8200 Aarhus N, Denmark; borre@clin.au.dk; 4 Department of Clinical Genetics, Aarhus University Hospital, 8200 Aarhus N, Denmark; else.marie.vestergaard@clin.au.dk; 5 Institute of Surgical Pathology, University Hospital Zurich, 8091 Zurich, Switzerland; peter.wild@usz.ch

**Keywords:** prostate cancer, DNA methylation, expression, TFF3, biomarker, diagnosis, prognosis

## Abstract

Overdiagnosis and overtreatment of clinically insignificant tumors remains a major problem in prostate cancer (PC) due to suboptimal diagnostic and prognostic tools. Thus, novel biomarkers are urgently needed. In this study, we investigated the biomarker potential of Trefoil factor 3 (*TFF3*) promoter methylation and RNA expression levels for PC. Initially, by quantitative methylation specific PCR (qMSP) analysis of a large radical prostatectomy (RP) cohort (*n* = 292), we found that the *TFF3* promoter was significantly hypomethylated in PC compared to non-malignant (NM) prostate tissue samples (*p* < 0.001) with an AUC (area under the curve) of 0.908 by receiver operating characteristics (ROC) curve analysis. Moreover, significant *TFF3* promoter hypomethylation (*p* ≤ 0.010) as well as overexpression (*p* < 0.001) was found in PC samples from another large independent patient sample set (498 PC vs. 67 NM) analyzed by Illumina 450K DNA methylation arrays and/or RNA sequencing. *TFF3* promoter methylation and transcriptional expression levels were inversely correlated, suggesting that epigenetic mechanisms contribute to the regulation of gene activity. Furthermore, low TFF3 expression was significantly associated with high ERG, ETS transcription factor (ERG) expression (*p* < 0.001), as well as with high Gleason score (*p* < 0.001), advanced pathological T-stage (*p* < 0.001), and prostate-specific antigen (PSA) recurrence after RP (*p* = 0.013; univariate Cox regression analysis). There were no significant associations between *TFF3* promoter methylation levels, ERG status, or PSA recurrence in these RP cohorts. In conclusion, our results demonstrated diagnostic biomarker potential of *TFF3* promoter hypomethylation for PC as well as prognostic biomarker potential of TFF3 RNA expression. To the best of our knowledge, this is the most comprehensive study of *TFF3* promoter methylation and transcriptional expression in PC to date.

## 1. Introduction

Prostate cancer (PC) is the most common non-skin cancer and the third most lethal malignancy amongst European men [1]. Some PCs remain latent and cause no significant symptoms or risk of morbidity within the lifetime of the patients, while other PCs progress to aggressive metastatic disease. Localized PC is curable by radical prostatectomy (RP) or radiation therapy, but only palliative treatments are available for metastatic PC. Therefore, early detection of PC is crucial. However, currently available diagnostic and prognostic tools for PC are suboptimal resulting in overdiagnosis and overtreatment of many clinically insignificant PCs [2]. Thus, there is an urgent need for novel diagnostic and prognostic biomarkers for PC.

Aberrant DNA hypermethylation of CpG island-containing gene promoters is a hallmark for PC and other malignancies, whereas non-CpG island promoters may become either hyper- or hypomethylated in cancer cells [3]. Some of the genes affected by perturbed promoter methylation levels are potential tumor suppressors or drivers of PC, as their transcriptional expression is repressed or activated upon promoter hyper- or hypomethylation, respectively [4]. In recent years, several candidate promoter hypermethylation markers for PC diagnosis have been identified [5,6,7,8,9,10], some of which have also shown promising prognostic potential for prediction of prostate-specific antigen (PSA) recurrence after RP [5,6,8,11,12,13,14]. Likewise, aberrant promoter hypomethylation has been proposed as a cancer biomarker in, e.g., myelofibrosis [15] and glioma [16], but it has not been extensively studied for PC.

The Trefoil factor 3 (*TFF3*) gene has a non-CpG island promoter and encodes the TFF3 protein, also known as intestinal trefoil factor, which is part of the mammalian family of trefoil factors (TFF1–3) [17]. TFF3 is a small secreted peptide that is present in almost all mucin-secreting tissues, but is most abundant in goblet cells of the gastrointestinal tract, where it protects the epithelial barrier by stimulation of mucosal restitution and inhibition of apoptosis [18,19,20,21]. In relation to cancer, TFF3 has been proposed to function both as an oncogene and as a tumor suppressor. Thus, high TFF3 expression has been associated with favorable prognosis in ovarian cancer [22] and with low grade in early stage breast cancer [23], and TFF3 seems to act as a tumor suppressor in thyroid cancer [24]. In contrast, TFF3 protein overexpression has been linked with aggressive disease in colon, gastric, and mammary cancer [25,26,27,28]. Likewise, overexpression studies in cell line models have suggested that *TFF3* serves as an oncogene in advanced metastatic castration-resistant PC [29]. However, three independent tissue microarray (TMA) studies of early stage hormone-naive PC (*n* = 268, *n* = 235, and *n* = 96 RP samples, respectively) showed no significant association between TFF3 protein levels and PSA recurrence after RP [30,31,32]. Furthermore, TFF3 protein expression has been reported to be significantly up-regulated only in the subset of PCs that are negative for the *TMPRRS2*-*ERG* (transmembrane protease, serine 2-ERG, ETS-transcription factor) gene fusion, which occurs in approximately 50% of primary PCs [33,34], potentially further complicating the possible association of TFF3 protein levels with prognosis as ERG fusion status is not clearly associated with PC outcome after RP [35].

Although we have previously reported that the *TFF3* promoter is hypomethylated in PC tissue samples, this was based only on a small patient sample set (10 PC vs. 12 benign prostatic hyperplasia (BPH) tissue samples) [36]. In the same study, we showed that *TFF3* promoter methylation and RNA expression levels were inversely correlated in a small set of prostate (cancer) cell lines, suggesting epigenetic regulation of gene activity [36]. However, larger patient sample sets are needed to investigate the diagnostic and prognostic biomarker potential of *TFF3* promoter methylation and RNA expression in PC.

Accordingly, in the present study, we have evaluated *TFF3* promoter hypomethylation and RNA expression in multiple large PC patient cohorts. First, using quantitative methylation specific PCR (qMSP) analysis, we found highly frequent and cancer-specific *TFF3* promoter hypomethylation in a set of 292 PC compared to 33 non-malignant (NM) prostate tissue samples. This was validated in an independent patient sample set comprising 497 PC and 50 NM prostate tissue samples, analyzed on the Illumina 450K DNA methylation array (450K). Using matched RNA sequencing (RNAseq) data, we also found that TFF3 RNA levels were significantly upregulated in PC compared to NM prostate tissue samples as well as significantly inversely correlated with promoter methylation levels, consistent with epigenetic regulation of *TFF3* gene activity. Moreover, low TFF3 RNA expression was significantly associated with high ERG expression, high Gleason score, advanced pathological T-stage, and PSA recurrence after RP. In contrast, there was no significant association between *TFF3* promoter methylation levels, ERG status, and PSA recurrence risk in these RP cohorts.

## 2. Results

### 2.1. Hypomethylation of the Trefoil Factor 3 (TFF3) Promoter Region in Prostate Cancer (PC) Samples

**Figure 1:**
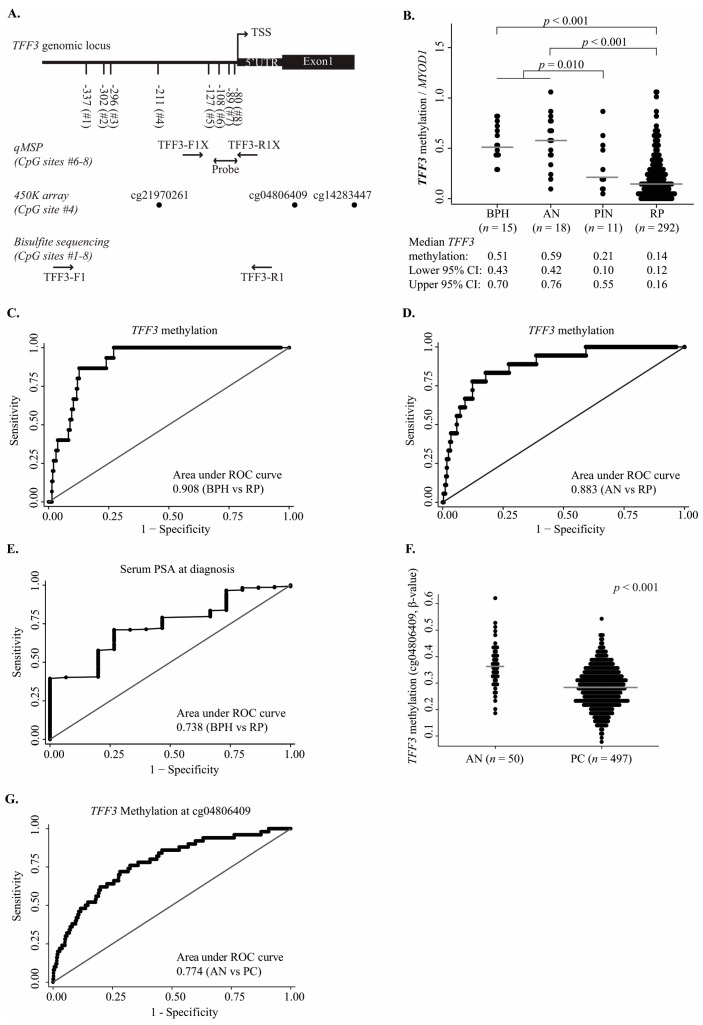
Hypomethylation of trefoil factor 3 (*TFF3*) in prostate cancer (PC) samples. (**A**) Schematic structure of the promoter region of *TFF3* including eight CpG sites (CpG sites #1–8), 5′ UTR, transcription start site (TSS), and exon 1. Regions analyzed by qMSP (CpG sites Nos. 6–8) and bisulfite sequencing (CpG sites #1–8) are marked with arrows, indicating primer/probe positions. The positions of the Illumina 450K probes (cg21970261 (CpG site No. 4), cg04806409, and cg14283447) are indicated with black dots; (**B**) *TFF3* methylation quantified by qMSP in BPH, AN, PIN, and RP samples. Median methylation and 95% confidence intervals (CI) are indicated; (**C**) ROC curve analysis of cancer specificity of *TFF3* promoter methylation in BPH vs. RP samples; (**D**) ROC curve analysis of cancer specificity of *TFF3* promoter methylation in AN vs. RP samples; (**E**) ROC curve analysis of cancer specificity of serum PSA levels at diagnosis in BPH vs. RP samples; (**F**) Promoter methylation of *TFF3* in 450K data from TCGA (cg04806409) in AN (*n* = 50) vs. PC (*n* = 497) samples; (**G**) ROC curve analysis of cancer specificity of *TFF3* promoter methylation in TCGA AN vs. PC samples for Illumina CpG site cg04806409. Abbreviations: 450K, Illumina 450K DNA methylation array; UTR, untranslated region; qMSP, quantitative methylation specific PCR; BPH, benign prostatic hyperplasia; AN, adjacent normal; PIN, prostate intraepithelial neoplasia; RP, radical prostatectomy; *p*, *p*-value (Mann–Whitney *U* test); Grey line, median methylation.

To investigate the diagnostic potential of *TFF3* promoter hypomethylation, we used qMSP to analyze *TFF3* promoter methylation levels in 15 BPH, 18 adjacent normal (AN), 11 prostate intraepithelial neoplasia (PIN), and 292 PC tissue samples from a large RP cohort with long clinical follow-up (Table 1, Figure 1A). The qMSP assay was designed to cover the most frequently hypomethylated region of the *TFF3* promoter (CpG sites Nos. 6–8), as identified previously by bisulfite sequencing [36]. *TFF3* was significantly hypomethylated in RP (PC) compared to BPH and AN samples (*p* < 0.001, Figure 1B), whereas no significant difference in methylation levels were observed between BPH and AN samples (*p* = 0.800, Figure 1B). Pre-malignant PIN lesions were significantly hypomethylated compared to non-malignant (BPH and AN) prostate tissue samples (*p* = 0.010, Figure 1B), suggesting that loss of *TFF3* promoter methylation may be an early event in prostate carcinogenesis.

By receiver operating characteristics (ROC) curve analysis, *TFF3* promoter hypomethylation was highly cancer-specific when comparing RP to BPH samples (area under the curve (AUC) 0.908, Figure 1C) and to AN samples (AUC 0.883, Figure 1D). Furthermore, at a sensitivity of 86.7%, the specificity of *TFF3* hypomethylation for RP vs. BPH samples was 87.3%, and at a sensitivity of 83.3% for RP vs. AN samples the specificity was 82.1%. These results are similar to the AUC values, sensitivities, and specificities previously reported for promoter hypermethylation marker candidates for PC diagnosis [5,6,8,37]. In comparison, serum PSA only showed an AUC of 0.738 for distinguishing RP and BPH patients in our cohort (Figure 1E). Thus, the *TFF3* promoter was frequently hypomethylated in PC and loss of *TFF3* promoter methylation was highly cancer-specific in this RP cohort, indicating promising diagnostic potential.

To validate the cancer-specific hypomethylation of *TFF3* in an independent RP patient cohort, we analyzed 450K data from TCGA for 497 PC and 50 AN tissue samples (Table 1) [38]. Out of nine CpG sites interrogated by probes on the 450K array and annotated to the *TFF3* gene, three were located near the qMSP assay (Illumina ID: cg21970261, cg04806409, and cg14283447, Figure 1A) and were significantly hypomethylated in PC compared to AN samples (*p* ≤ 0.010, Figure 1F and Figure S1A,B), corroborating our qMSP results (Figure 1B). None of the other six CpG sites/probes were significantly hypomethylated in PC samples. The CpG site with the largest difference in β-values between AN and PC samples was cg04806409, which was located downstream of the qMSP assay, and had an AUC of 0.774 (Figure 1G). We note, however, that the difference in median methylation levels between PC and AN samples (Figure 1F and Figure S1A,B) as well as the corresponding AUC values (0.610–0.774, Figure 1G and Figure S1C,D were smaller for the Illumina sites compared to CpG sites Nos. 6–8 (Figure 1B,D). This is in agreement with our previous bisulfite sequencing results that also showed CpG sites Nos. 6–8 to be more frequently hypomethylated in PC tissue samples than the upstream CpG site No. 4 [36], and together demonstrating that the methylation status of the *TFF3* promoter region is CpG site dependent.

In summary, we here report significant cancer-specific hypomethylation of the *TFF3* promoter region in two independent patient cohorts, including a total of 787 PC and 155 NM prostate tissue samples.

### 2.2. Correlation between TFF3 Promoter Methylation, Clinicopathological Parameters, and ERG, ETS Transcription Factor (ERG) Status

To assess the association between *TFF3* promoter methylation levels and PC aggressiveness, we compared DNA methylation levels with standard clinicopathological variables: age at diagnosis, pre-operative PSA level, Gleason score, pathological tumor (pT)-stage, pathological lymph node (pN)-stage, surgical margin, and PSA recurrence status in our large RP cohort (*n* = 292; Table 1). We found a weak, but statistically significant association between *TFF3* promoter hypomethylation at CpG sites Nos. 6–8 and high pathological Gleason score (odds ratio from logistic regression = 0.264, *p* < 0.001, Table 2; Mann–Whitney *U* test *p* = 0.007, Figure S2A), while there were no significant associations with any of the other clinicopathological parameters (Figure S2B–G, Table 2).

Using a subset of samples from these RP patients (*n* = 102; Table 1) previously analyzed on a TMA and scored for ERG immunoreactivity [39], we found no significant association between *TFF3* promoter methylation at CpG sites Nos. 6–8 and ERG status (Figure S2H; *p* = 0.578, Mann–Whitney *U* test). In the TCGA patient set, there was a significant difference in methylation levels between patients with high and low ERG RNA expression at cg04806409, but the difference in median β-value was negligible (*p* = 0.0065, Mann–Whitney *U* test, difference in median = 0.02), and we found no significant difference in *TFF3* promoter methylation at CpG sites cg21970261 and cg14283447. In summary, *TFF3* promoter methylation levels are likely not affected by ERG expression levels.

### 2.3. Survival Analysis

Next, we tested the prognostic potential of *TFF3* promoter methylation for prediction of post-operative PSA recurrence risk in our RP cohort by univariate Cox regression analysis. High Gleason score, advanced pT-stage, positive lymph node, and positive surgical margin status were all significantly associated with early PSA recurrence (Table 3), indicating that this is a representative RP cohort. In contrast, no significant association was found between PSA recurrence-free survival and age at diagnosis, ERG status, or *TFF3* promoter methylation level at CpG sites Nos. 6–8, respectively (Table 3). Similarly, in the TCGA RP cohort (*n* = 389), the methylation level did not predict time to PSA recurrence at any of the three CpG sites, whereas high Gleason score and advanced pT-stage were significantly associated with PSA recurrence (Table 4). ERG status was not associated with time to PSA recurrence, neither in our RP cohort (Table 3) nor in the TCGA RP cohort (Table 4), consistent with previous reports [35].

### 2.4. TFF3 RNA Expression Patterns in Public Datasets for PC

**Figure 2:**
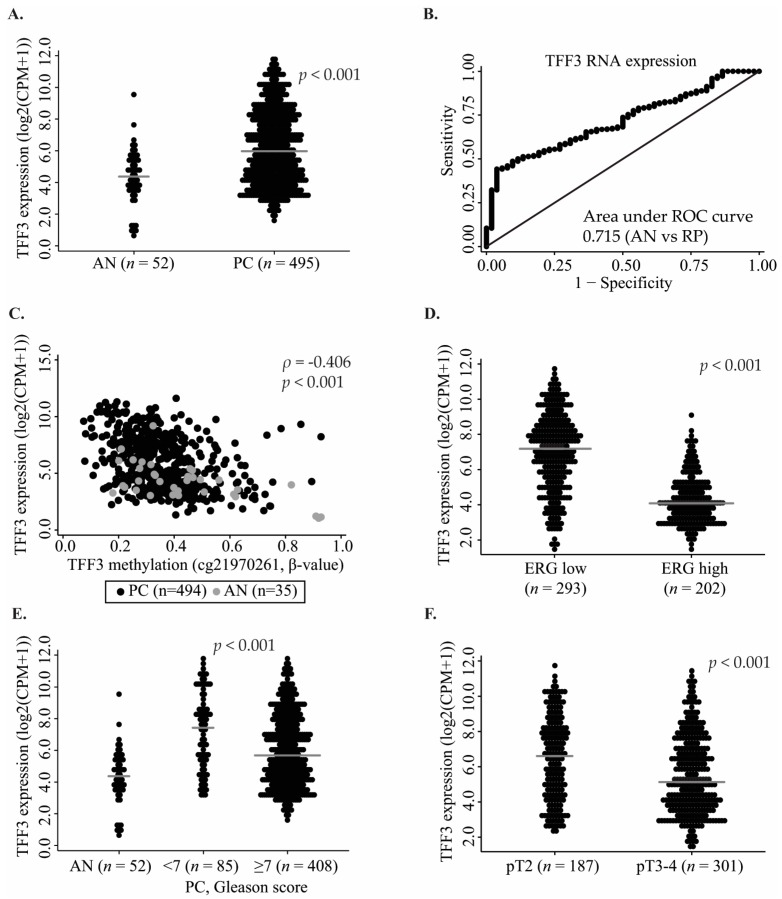
Transcriptional TFF3 expression based on RNAseq data from TCGA. (**A**) TFF3 RNA expression in AN (*n* = 52) and PC (*n* = 495) tissue samples; (**B**) ROC curve analysis for TFF3 RNA expression in AN vs. PC samples; (**C**) Correlation between *TFF3* promoter methylation (cg21970261; CpG site No. 4) and TFF3 RNA expression in AN (*n* = 35, grey) and PC (*n* = 494, black) samples from TCGA. Correlations between TFF3 RNA expression and (**D**) ERG RNA expression (*n* = 495), (**E**) Gleason score (*n* = 493), and (**F**) pathological T-stage (*n* = 488). Abbreviations: CPM, counts per million; AN, adjacent normal; PC, prostate cancer; *p*, *p*-value (Mann–Whitney *U* test or Spearman’s correlation test); ρ, Spearman’s rho; pT, pathological T-stage; Grey line, median expression.

Using RNAseq data from TCGA [38], we evaluated TFF3 transcriptional expression levels in 495 PC and 52 AN prostate tissue samples. TFF3 RNA was significantly upregulated in PC compared to AN samples (*p* < 0.001, Figure 2A), consistent with promoter hypomethylation in PC (Figure 1B,F). At the RNA level, TFF3 showed only moderate diagnostic biomarker potential by ROC curve analysis (AUC 0.715, Figure 2B). Furthermore, matched RNAseq and 450K data were available for 494 PC and 35 AN tissue samples [38] and demonstrated a significant inverse correlation between RNA expression and *TFF3* promoter methylation at two out of three CpG sites (cg21970261: Spearman’s ρ = −0.406, *p* < 0.001, Figure 2C; cg14283447: Spearman’s ρ = −0.295, *p* < 0.001, Figure S3B; cg04806409, *p* > 0.05, Figure S2A), thereby corroborating and expanding our previous findings in a small set of prostatic cell lines [36], and together suggesting that epigenetic changes are associated with TFF3 upregulation in PC.

The significant upregulation of TFF3 RNA expression in PC was confirmed in two additional patient sample sets, including 126 PC + 29 AN and 36 PC + 19 AN [40,41] tissue samples profiled on microarrays (*p* < 0.001 and *p* = 0.019; Figure S4A,B), and also had similar AUCs (0.727 and 0.716 by ROC curve analysis). Overexpression of TFF3 RNA was specific for ERG negative PC in all of the three independent patient sets (Figure 2D and Figure S4C,D), corresponding to previous findings at the TFF3 protein level [33,42] and consistent with previous reports that the *TFF3* gene is downregulated by ERG [42]. In further agreement with this, TFF3 and ERG RNA expression levels were significantly inversely correlated in all of the three public RNA expression datasets investigated (Spearman correlation test, TCGA: ρ = −0.604, *p* < 0.001, Taylor et al.: ρ = −0.697, *p* < 0.001; Mortensen et al.: ρ = −0.695, *p* < 0.001) [38,40,41].

### 2.5. Prognostic Potential of TFF3 RNA Expression

**Figure 3:**
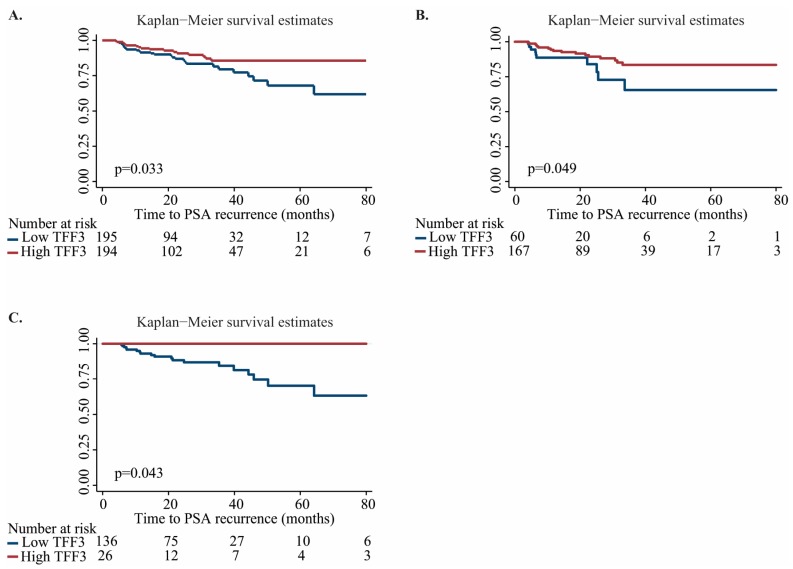
Kaplan–Meier analysis of PSA recurence-free survival based on TFF3 RNA expression levels (low vs. high) in TCGA PC samples. (**A**) all PC samples; (**B**) PC samples with low ERG RNA expression; and (**C**) PC samples with high ERG RNA expression. Blue line, low TFF3 RNA expression; Red line, high TFF3 RNA expression; *p*, *p*-value (log-rank test).

To evaluate whether TFF3 RNA levels were associated with PC aggressiveness, we tested the correlation between TFF3 RNA expression and clinicopathological variables in the large TCGA RP patient cohort (495 PC and 52 AN). Low TFF3 RNA expression was significantly associated with high Gleason score and with advanced pathological T-stage (*p* < 0.001, Mann–Whitney *U* test; Figure 2E,F), whereas no significant association with pre-operative PSA level (*p* = 0.829; Spearman correlation test) or surgical margin status (*p* = 0.151; Mann–Whitney *U* test) was found (Figure S5A,B). In contrast, low TFF3 RNA expression was significantly associated with early PSA recurrence in univariate Cox regression analysis (HR 0.84 (0.74–0.97), *p* = 0.013, Table 4), suggesting that TFF3 RNA expression has prognostic biomarker potential for PC. Although TFF3 RNA expression did not remain significant after correction for Gleason score and pT-stage (*p* = 0.177; Table S1) in this patient cohort with relatively limited follow-up and few PSA recurrences (Table 1), Kaplan–Meier analyses showed that PC patients with low TFF3 expression had significantly increased risk of PSA recurrence (Figure 3A, *p* = 0.039, log-rank test).

ERG RNA expression levels did not have significant prognostic value for prediction of recurrence-free survival in the TCGA cohort (*p* = 0.741, univariate cox regression analysis; Table 4). However, when grouping PC patients according to ERG expression status (low or high), the prognostic potential of TFF3 expression remained significant in univariate cox regression analysis in both the ERG low (HR 0.79 (0.67–0.94), *p* = 0.008) and the ERG high subgroup (HR 0.84 (0.74–0.97), *p* = 0.013), which was also confirmed by Kaplan–Meier analysis (Figure 3B,C, *p* = 0.049 and *p* = 0.043, log-rank test). In conclusion, these results indicate that low transcriptional expression of TFF3 is a significant adverse predictor for PSA recurrence after prostatectomy in both ERG negative and ERG positive PC.

## 3. Discussion

To the best of our knowledge, this is the largest study of *TFF3* promoter methylation and RNA expression in PC to date. We found that the *TFF3* gene promoter was frequently hypomethylated in PC compared to non-malignant prostate tissue samples, suggesting diagnostic biomarker potential for PC. Additionally, TFF3 RNA expression levels were significantly increased in PC tissue samples and correlated inversely with *TFF3* promoter methylation, consistent with epigenetic regulation of gene activity. Furthermore, low TFF3 RNA levels were significantly associated with high ERG expression, high Gleason score, advanced pT-stage, and early PSA recurrence after RP. Together, our results demonstrate diagnostic biomarker potential of *TFF3* promoter hypomethylation as well as prognostic biomarker potential of TFF3 RNA expression for clinically localized PC.

We assessed the diagnostic biomarker potential of *TFF3* at the promoter methylation as well as RNA expression level in multiple large RP patient cohorts. Whereas RNA expression and promoter methylation at the 450K CpG sites in the *TFF3* promoter had moderate diagnostic value (AUC range: 0.610–0.774), hypomethylation of CpG sites Nos. 6–8 was highly cancer-specific (AUC 0.908; BPH vs. RP) and superior to serum PSA in our patient cohort (AUC 0.738). The diagnostic accuracy (AUC value) demonstrated in the present study for *TFF3* promoter hypomethylation at CpG sites Nos. 6–8 is similar to that of previously reported candidate hypermethylation markers for PC [5,6,8,9,10], including one of the most well-described methylation markers *GSTP1* [43,44]. Furthermore, the results of this large-scale study expand and confirm our previous finding of *TFF3* promoter hypomethylation in PC, which was based only on a small patient sample set (10 PC vs. 12 BPH) [36].

The relatively high rate of false-negative prostate biopsies remains a major clinical challenge for PC diagnosis and results in many repeat biopsies [45,46]. As the biopsy procedure is associated with considerable risk of sepsis [47], unnecessary biopsies should be avoided. Thus, it would be of potential future clinical relevance to investigate the possible existence of PC-associated *TFF3* hypomethylation field effects in morphologically non-malignant prostate needle biopsies, which in turn might be used to predict the need for repeat biopsy. The existence of epigenetic cancer field effects in relation to PC has previously been reported for a number of aberrantly hypermethylated genes [48,49,50,51], but further studies are needed to investigate if this is also the case for *TFF3* promoter hypomethylation. Moreover, non/minimally-invasive biomarkers that can accurately predict the need for initial/repeat prostate biopsy are still lacking. Accordingly, future studies should also investigate the diagnostic biomarker potential of *TFF3* hypomethylation in plasma and urine samples.

Of note, in addition to PC samples, *TFF3* was also hypomethylated in a small set of PIN samples (*n* = 11). At present, it is not clear how men with high grade PIN should be treated, however, current recommendations suggest that men included in screening studies should undergo re-biopsy within six months after a diagnosis of multifocal high-grade PIN [52]. Thus, it is possible, that biomarkers reporting PIN as well as PC, such as *TFF3* hypomethylation, may be advantageous, either alone or in combination with PC specific biomarkers.

In the present study, TFF3 RNA levels were significantly increased in 495 PC compared to 52 benign tissue samples. Furthermore, this is the first study to demonstrate a significant association between low TFF3 RNA levels in PC samples and high pT-stage (*n* = 488), high Gleason score (*n* = 493), and PSA recurrence after RP (*n* = 389). In agreement with our results, TFF3 protein was previously reported to be overexpressed in malignant compared to benign prostate tissue samples in three TMA studies, including 268, 235, and 96 RP samples, respectively [30,31,32]. Moreover, TFF3 immunoreactivity was significantly lower in high-stage than low-stage PC in one of these studies [30] and a similar trend was observed in another study [32], consistent with our results. However, in contrast to our findings at the transcriptional level, none of the previous TMA studies found significant associations between PSA recurrence after RP and TFF3 protein IHC scores. Differences in the exact composition and size of RP cohorts used may explain these seeming discrepancies in prognostic potential for TFF3 at the transcriptional and protein expression level. In addition, median follow-up times were not reported in the TMA studies [30,31,32], potentially compromising the interpretation of patient outcome results. The reported differences in prognostic potential of TFF3 protein IHC scores and RNA levels for PC might also be explained by the use of different methodologies. IHC scores are semi-quantitative and lack dynamic range compared to RNAseq, which offers a quantitative digital measurement of RNA levels. Moreover, post-transcriptional regulatory mechanisms as well as secretion of TFF3 protein [53] could make a direct comparison between TFF3 RNA and protein expression levels problematic. Lastly, we would suggest that cellular localization of TFF3 protein should be analyzed together with expression levels, as localization has been suggested to impact the association between TFF3 protein and cancer aggressiveness in breast cancer [23]. Additional large PC patient cohorts should be analyzed for TFF3 RNA expression to further validate the prognostic value reported here for TFF3 at the transcriptional level. This should include cohorts with long clinical follow-up, to assess if TFF3 RNA levels could have independent prognostic value.

In this study, TFF3 and ERG transcriptional expression levels in PC tissue samples were significantly inversely correlated, as also previously reported for hormone-naive PC [31,42]. Notably, the prognostic potential of TFF3 RNA expression for post-operative PSA recurrence remained significant in both subgroups, suggesting that it is independent of ERG status. While this is the first report to investigate the prognostic potential of TFF3 expression in patients stratified according to ERG status (RNA levels low or high), further studies are needed to confirm our findings.

Currently, many patients with low-stage/low-grade PC are believed to be over-diagnosed and over-treated [54]. New prognostic biomarkers may be used to stratify these patients into high- and low-risk subgroups in order to guide treatment selection [55]. Thus, future studies should evaluate whether TFF3 RNA levels in prostate biopsies can be used to stratify low-intermediate risk PC patients for e.g., active surveillance or RP at the time of diagnosis. In such future studies, it would also be relevant to compare the prognostic value of TFF3 RNA expression to previously published RNA expression signatures associated with aggressiveness of early-stage PC [56] and to determine if TFF3 can improve the prognostic value of such signatures.

In the present study, we identified significant associations between low TFF3 RNA levels and high Gleason score, advanced pT-stage, and early PSA recurrence after RP. Thus, our results indicate that low TFF3 RNA expression is an adverse prognostic factor in PC. This might appear to contradict previous functional studies in PC cell lines (cells isolated from metastatic disease), where high TFF3 expression has been suggested to have an oncogenic role in late stage PC [29]. However, other reports indicate that TFF3 may function as an oncogene or as a tumor suppressor depending on the cellular context. Thus, in breast cancer, high TFF3 protein expression has been associated with low grade disease in early stages, but with aggressive disease in advanced stages [23]. Furthermore, in gastric cancer, high TFF3 protein levels have been associated with reduced overall survival only in lymph-node positive and highly undifferentiated tumors [26], suggesting that high TFF3 protein levels are associated with aggressiveness specifically in advanced gastric cancer. Further underlining that TFF3 can function as an oncogene or a tumor suppressor depending on the cell type, previously reported functional studies have shown that TFF3 overexpression promoted proliferation in a breast cancer cell line, but inhibited proliferation in a thyroid cancer cell line [24]. Furthermore, whereas high TFF3 protein levels in colon cancer have been associated with early recurrence after surgery [25], high TFF3 expression in ovarian cancer is associated with longer recurrence-free survival [22]. Thus, *TFF3* may serve oncogenic or tumor suppressive functions depending on cell type and disease stage in at least some malignancies. The different functional roles of TFF3 have been proposed to be related to the polarity of TFF3 secretion from the cells [23]. Thus, well-differentiated tumors may retain cell polarity allowing TFF3 to be secreted from the apical epithelial cell surface into, e.g., the lumen of glandular tissues, whereas in poorly differentiated tumors, TFF3 may be secreted into the stroma, where it can stimulate cell proliferation and migration, as shown in functional studies in cell line models of advanced PC [29]. Further studies are needed to investigate this in PC.

In conclusion, we have demonstrated highly significant and frequent cancer-specific promoter hypomethylation of *TFF3* in malignant compared to non-malignant prostate tissue samples in two large independent RP cohorts, including a total of 789 PC and 94 NM tissue samples. Furthermore, we found a significant inverse correlation between DNA methylation and RNA expression levels for *TFF3*, indicating epigenetic regulation of *TFF3* gene activity. Moreover, low TFF3 RNA expression was significantly associated with early PSA recurrence after RP, suggesting that TFF3 RNA expression has prognostic biomarker potential for PC. This is the largest study to date investigating the promoter methylation and RNA expression of *TFF3* in PC.

## 4. Materials and Methods

### 4.1. Patient Material

The RP cohort used for quantitative methylation specific PCR (qMSP) consisted of consecutive curatively intended RPs of histologically verified clinically localized prostate cancer (PC), as previously described [5,6,8].

In brief, formalin-fixed paraffin-embedded (FFPE) RP samples were collected in Denmark at Department of Urology, Aarhus University Hospital from 1997 to 2005, and in Switzerland at University Hospital Zurich from 1993 to 2001. Hematoxylin and eosin (HE) stained slides from each patient was evaluated by a trained pathologist, and punch biopsies of 1.5 mm from the corresponding FFPE blocks were attained from representative regions with more than 90% tumor content. Patients with pre/post-endocrine treatment (*n* = 40) or lack of follow-up (*n* = 56) and samples with a poor DNA quality (*n* = 69) were omitted from the study. The final analysis included 292 RP patients (Table 1).

In addition, samples of benign prostatic hyperplasia (BPH, *n* = 15), adjacent normal (AN, *n* = 18), and prostate intraepithelial neoplasia (PIN, *n* = 11) were included (Table 1), as described previously [5,6,8]. Briefly, AN and PIN samples were obtained by punch biopsy of FFPE RP specimens, whereas BPH samples were obtained from FFPE tissue from transurethral resections of the prostate. The Danish RP samples were previously used for generation of a tissue microarray (TMA) [8,39], and were classified as ERG positive or negative based on ERG immunohistochemistry scores [39], which are known to closely reflect ERG fusion status [57,58].

### 4.2. Quantitative Methylation Specific PCR (qMSP)

For the FFPE samples collected in Denmark, DNA was extracted with the gDNA Eliminator columns from the miRNeasy FFPE Kit (Qiagen, Hilden, Germany), and for FFPE samples collected in Switzerland, DNA was extracted with the the Blood and Cell Culture DNA Kit (Qiagen). The EZ-96 DNA Methylation-Gold Kit^TM^ (Zymo, Irvine, CA, USA) was used for bisulfite conversion of extracted genomic DNA, as previously described [5,6,8].

The probe and primers (Table S2) used for qMSP, targeted the promoter region of *TFF3*. Furthermore, a *MYOD1* assay targeting a genomic region without CpG sites was used as a control assay, as previously described [5]. Triplicate qMSP reactions (5 μL) was run on each patient sample, on standard curves of serially diluted methylated DNA, and on bisulfite converted and un-converted CpGenome Universal Methylated and Unmethylated DNA (Millipore, Billerica, MA, USA) as controls. A total of 5 ng bisulfite converted DNA was used as input, and reactions included 3 pmol of each primer and 1 pmol probe as well as Taqman universal Mastermix no UNG (Applied Biosystems, Foster City, CA, USA). Reactions were pippeted using the Biomek NXP Laboratory Automation Workstation and run on the 7900 HT real time thermal cycler (Applied Biosystems): 2 min at 50 °C, 10 min at 95 °C, and 50 cycles of 15 s at 95 °C and 1 min at 56 °C. *TFF3* and *MYOD1* quantities were estimated from the standard curve using SDS 2.4 software (Applied Biosystems), and *TFF3* methylation was normalized to *MYOD1* to control for DNA input. Samples were excluded from the analysis if two out of three *C*_t_ values for *MYOD1* exceeded 36. Furthermore, outliers that were more than 2 *C*_t_ values lower/higher than the other *C*_t_ values were removed.

Bisulfite sequencing results from seven prostate cell lines (LNCaP, VCaP, DuCaP, PC3, BPH1, DU145, and PNT1A) from our previous study [36], were used to test the specificity of our qMSP assay. PCR primers used for bisulfite sequencing are listed in Table S2. We found that the qMSP assay reported fully methylated alleles accurately but slightly underestimated methylation levels of heterogeneously methylated DNA (Pearson: 0.87, *p* = 0.005, Figure S6A,B, compare, e.g., methylation levels of LNCaP, VCaP, and DUCaP).

### 4.3. Microarray and RNAseq Data

Illumina 450K DNA methylation array (450K, 497 RP and 50 matched AN samples), RNA sequencing (RNAseq, 495 RP and 52 matched AN samples), and clinical data were downloaded from The Cancer Genome Atlas (TCGA, http://cancergenome.nih.gov/) (Table 1) [38]. The 450K data were peak corrected, as previously described [59], and the methylation level for each CpG site was given as a β-value (ranging from 0 to 1). RNAseq data from TCGA were mapped to the human genome (hg19) using Tophat [60] and the Bowtie aligner [61], and HTSeq [62] was used to summarize reads per gene. Gene expression quantified by RNAseq is given as counts per million (CPM).

Furthermore, normalized microarray RNA expression data and clinical data were downloaded for 126 PC samples and 29 matched AN samples from GEO (GSE21034; Affymetrix Human Exon 1.0 ST array) [40] and for 36 PC and 14 normal prostate samples from GEO (GSE46602; Affymetrix U133 2.0 Plus microarray) [41].

### 4.4. Statistical Analysis

All statistical analyses were performed in STATA v. 13.1 (STATA, College Station, TX, USA). Mann–Whitney *U* tests, ROC analyses, two-sided log-rank tests, univariate Cox regression analyses and/or Kaplan–Meier analysis, using PSA recurrence (cutoff ≥ 0.2 ng/mL) as endpoint, were used to investigate the diagnostic and prognostic potential of *TFF3* promoter methylation and RNA expression. For patients that had not experienced PSA recurrence, the last normal PSA measurement was used as endpoint. Pearson correlations were used to compare the performance of the designed qMSP assay to previous bisulfite sequencing results [36], Spearman correlations were used to examine the correlation between *TFF3* promoter methylation and RNA expression, and Mann–Whitney *U* tests were used to investigate the association between *TFF3* promoter methylation and RNA expression to clinicopathological parameters. For all PC samples in the TCGA RNAseq data, TFF3 RNA expression was dichotomized separating the patients into two equally sized subgroups (low and high TFF3 expression), as ROC curve analysis did not result in an obvious cutoff. Statistical significance in Kaplan–Meier analysis was calculated using two-sided log-rank tests.

### 4.5. Ethical Approval

The study was approved in Switzerland by the Ethical Committee of the Canton of Zurich under approval numbers KEK-ZH-No. 2007-0025 and KEK-ZH-No. 2014-0604, and in Denmark by The Central Denmark Region Committees on Health Research Ethics under approval number 2000/0299, and the Data Protection agency approval number 2013-41-2041.

## Figures and Tables

**Table 1:** Clinicopathologic characteristics of patient samples. For 102 of the patients analyzed by qMSP, ERG status was available from previous IHC analyses. Abbreviations: PC, prostate cancer samples from radical prostatectomies; AN, adjacent normal; BPH, benign prostatic hyperplasia; PIN, prostate intraepithelial neoplasia; pT, pathological tumor stage; Pre-op, preoperative; pN, pathological lymph node stage; IHC, immunohistochemistry; 450K, Illumina 450K DNA methylation array; qMSP, quantitative methylation specific PCR; RNAseq, RNA sequencing; ERG, ERG, ETS transcription factor; PSA, prostate-specific antigen.

Variable	PC Samples Analyzed by qMSP (*n* = 292)	PC Samples Analyzed by 450K and/or RNAseq (*n* = 498)
**Age (years)**		
Median (range)	63 (46–73)	61 (41–78)
**pT-stage**		
pT2	183 (62.7%)	188 (37.8%)
pT3	107 (36.6%)	293 (58.8%)
pT4	2 (0.7%)	10 (2.0%)
Unknown	0 (0%)	7 (1.4%)
**Gleason score**		
<7	113 (38.7%)	86 (17.3%)
7	142 (48.6%)	240 (48.2%)
>7	36 (12.3%)	170 (34.1%)
Unknown	1 (0.3%)	2 (0.4%)
**Pre-op. serum PSA (ng/mL)**		
Median (range)	11.8 (0.6–64.2)	7.5 (0.7–107)
0–10	114 (39.0%)	331 (66.5%)
>10	177 (60.1%)	152 (30.5%)
Unknown	1 (0.3%)	15 (3.0%)
**pN-stage**		
pN0	252 (86.3%)	0 (0.0%)
pN1	5 (1.7%)	0 (0.0%)
Unknown	35 (12.0%)	498 (100.0%)
**Surgical margin status**		
Negative	196 (57.9%)	316 (63.5%)
Positive	92 (31.5%)	152 (30.5%)
Unknown	4 (1.4%)	30 (6.0%)
**PSA recurrence**		
Yes	132 (45.2%)	46 (9.2%)
No	160 (54.8%)	346 (69.5%)
Unknown	0 (0.0%)	106 (21.3%)
**Follow-up (months)**		
Median (range)	65 (5–151)	20 (3–154)
**ERG status (IHC)**		
Pos	59 (20.2%)	0 (0.0%)
Neg	43 (14.7%)	0 (0.0%)
Unknown	190 (65.1%)	495 (100.0%)
**Non/pre-malignant samples**	**Age/years**	**Age/years**
	Median (range)	Median (range)
AN (*n* = 18, *n* = 67)	62 (56–72)	61 (43–72)
BPH (*n* = 15)	70 (56–83)	-
PIN (*n* = 11)	63 (54–68)	-

**Table 2:** Correlation between *TFF3* promoter methylation (CpG sites Nos. 6–8) and clinicopathological variables in RP samples (*n* = 292). Abbreviations: OR, odds ratio; CI, confidence interval; * *p*-value < 0.01.

Variable	Logistic Regression		Variable Value (Dichotomized)	Median *TFF3* Methylation (95% CI)	Mann–Whitney *p*-Value
	OR	*p*-Value			
Age	0.999	0.992	-	-	-
Pre-operative PSA	0.958	0.053	0–10 ng/mL	0.13 (0.10–0.15)	0.202
			>10 ng/mL	0.15 (0.13–0.20)	
Gleason score	0.264	<0.001 *	GS < 7	0.16 (0.13–0.22)	0.007 *
			GS ≥ 7	0.13 (0.11–0.15)	
Pathological T-stage	-	-	pT2	0.14 (0.12–0.19)	0.331
			pT3–4	0.13 (0.11–0.17)	
Pathological N-stage	-	-	pN0	0.15 (0.13–0.19)	0.367
			pN1	0.10 (0.00–0.44)	
Margin status	-	-	Neg	0.15 (0.12–0.19)	0.261
			Pos	0.14 (0.11–0.19)	
PSA recurrence status	-	-	No	0.13 (0.10–0.15)	0.258
			Yes	0.16 (0.13–0.21)	
ERG status (IHC)	-	-	Neg	0.22 (0.16–0.30)	0.578
			Pos	0.22 (0.15–0.28)	

**Table 3:** Cox regression analysis of PSA recurrence-free survival after radical prostatectomy in our PC cohort (*n* = 292). *TFF3* methylation for CpG sites Nos. 6–8. Abbreviations: Path, pathological; T, tumor; N, lymph node; Pre-op, preoperative; HR, hazard ratio; CI, confidence interval; * *p*-value < 0.005.

Variable		HR (95% CI)	*p*-Value
*TFF3* methylation	Cont.	1.01 (0.44–2.29)	0.986
Age	Cont.	0.98 (0.95–1.01)	0.136
Pre-op. PSA	Cont.	1.05 (1.03–1.06)	<0.001 *
Gleason score	<7 vs. ≥7	2.19 (1.46–3.28)	<0.001 *
Path. T-stage	pT2 vs. pT3–4	3.64 (2.58–5.12)	<0.001 *
Path. N-stage	pN0 vs. pN1	4.00 (1.62–9.85)	0.003 *
Surgical margin status	Neg vs. pos	3.28 (2.32–4.63)	<0.001 *
ERG status (IHC)	Neg vs. pos	1.13 (0.71–1.82)	0.606

**Table 4:** Cox regression analysis of PSA recurrence-free survival after radical prostatectomy in the TCGA PC patient cohort (*n* = 389). *TFF3* methylation for CpG site No. 4. Abbreviations: Path, pathological; T, tumor; Pre-op, preoperative; HR, hazard ratio; CI, confidence interval; * *p*-value < 0.05.

Variable		HR (95% CI)	*p*-Value
*TFF3* methylation, cg21970261	Cont.	1.96 (0.28–13.75)	0.497
*TFF3* methylation, cg04806409	Cont.	0.04 (0.00–1.79)	0.096
*TFF3* methylation, cg14283447	Cont.	0.97 (0.03–31.95)	0.985
TFF3 RNA expression	Cont.	0.84 (0.74–0.97)	0.013 *
Age	Cont.	1.02 (0.97–1.06)	0.479
Pre-op. PSA	Cont.	1.02 (1.00–1.04)	0.065
Path. Gleason score	<7 vs. ≥7	4.75 (1.15–19.61)	0.031 *
Path. T-stage	pT2 vs. pT3–4	6.02 (2.16–16.81)	0.001 *
Surgical margin status	Neg vs. pos	1.47 (0.82–2.65)	0.198
ERG RNA expression	Low vs. high	0.95 (0.50–1.63)	0.741

## Supplementary Materials

Supplementary materials can be found at www.mdpi.com/1422-0067/18/9/2017/s1.

## Abbreviations

450K	Illumina 450K DNA methylation array
AN	Adjacent normal
AUC	Area under the curve
BPH	Benign prostatic hyperplasia
CI	Confidence interval
CPM	Count per million
FFPE	Formalin-fixed paraffin embedded
HE	Hematoxylin and eosin
HR	Hazard ratio
NM	Non-malignant
OR	Odds ratio
Path	Pathological
PC	Prostate cancer
PIN	Prostatic intraepithelial neoplasia
PSA	Prostate specific antigen
qMSP	Quantitative methylation specific PCR
RNAseq	RNA sequencing
ROC	Receiver operating characteristics
RP	Radical prostatectomy
TCGA	The Cancer Genome Atlas
TFF3	Trefoil factor 3
TMA	Tissue microarray
