# Supplementary material for: Comprehensive Evaluation of TFF3 Promoter Hypomethylation and Molecular Biomarker Potential for Prostate Cancer Diagnosis and Prognosis
Source: Int J Mol Sci. 2017 Sep 20;18(9):2017. doi: 10.3390/ijms18092017 (PMC5618665; doi:10.3390/ijms18092017)
Supplement: Supplementary file 1 [file ijms-18-02017-s001.pdf]

Table S1: Multivariate Cox regression analysis of time to PSA recurrence in 392 TCGA PC samples. Only clinicopathological variables significant in univariate analysis and TFF3 RNA expression included.

**Variable**		**HR(95% CI)**	**p-value**
TFF3 RNA expression	Cont.	0.91 (0.79-1.04)	0.177
Path. Gleason score	<7 vs. ≥7	2.13 (0.48-9.36)	0.317
Path. T-stage	pT2 vs. pT3-4	4.77 (1.67-13.61)	**0.004***

Table S2: Primers and probes used for *TFF3* methylation analysis.

Analysis type	Primer/probe name	Sequence	Amplicon size	Input per 5μL reaction (pmol)
Bisulfite seq [1]	TFF3-F1	5′-AGGAAAGATAAGGAATTTTTGTGTTTT-3′	385-bp	
	TFF3-R1	5′-ACATACCTTTATCAAACTCCCAAAC-3′		
qMSP	TFF3-F1X	5′-AGGAGGGTAATTGATATATATT-3′	91-bp	3
	TFF3-R1X	5′-CCCACTATTTTAACAACAAAC-3′		3
	TFF3-P1M	FAM-5′-TCGGAATTAGAATTGGAATTCGTTTTTATCGT-3′-BHQ1	-	1
	MYOD1-F	5′-CCAACTCCAAATCCCCTCTCTAT-3′	106-bp	3
	MYOD1-R	5′-TGGTTTTTTTAGGGAGTAAGTTTGTT-3′		3
	MYOD1-P	FAM-5′-TCCCTTCCTATTCCTAAATCCAACCTAAATACCTCC-3′-BHQ1	-	1

1. Vestergaard, E.M.; Nexo, E.; Torring, N.; Borre, M.; Orntoft, T.F.; Sorensen, K.D. Promoter hypomethylation and upregulation of trefoil factors in prostate cancer. *Int J Cancer* **2010**, *127*, 1857-1865.

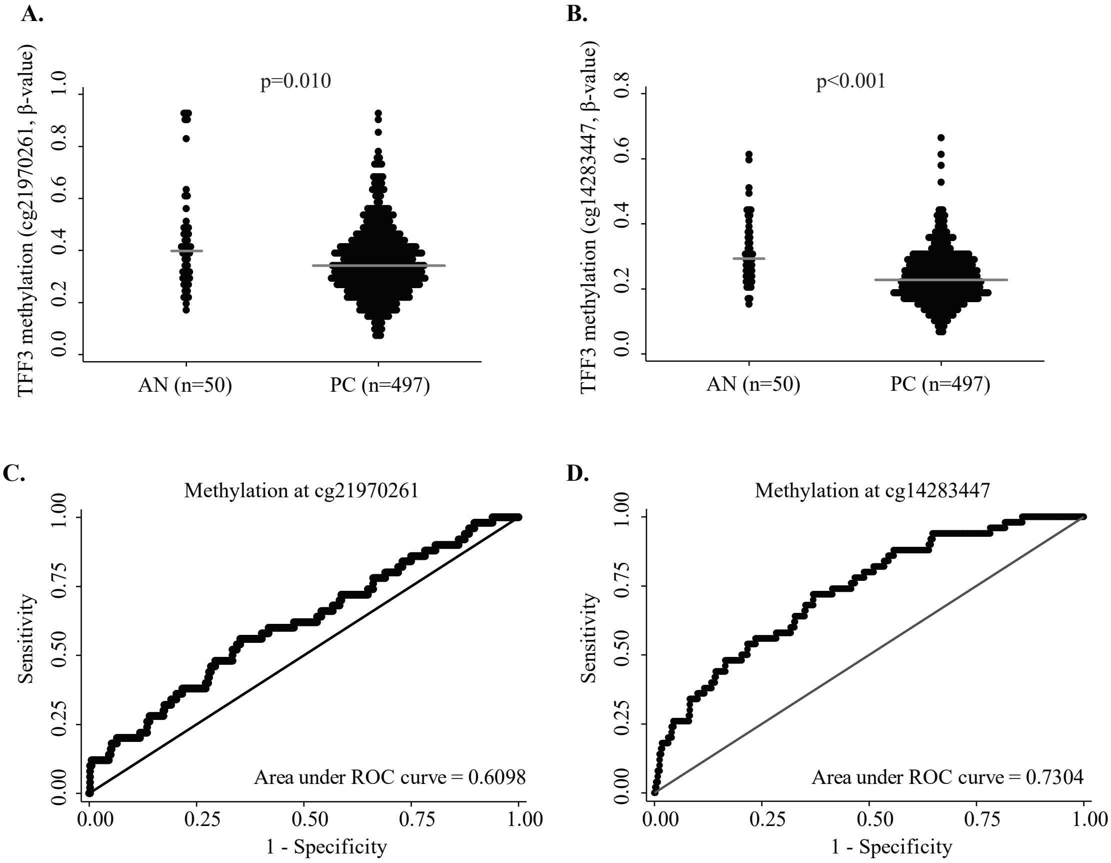

Figure S1: Hypomethylation of TFF3 in TCGA PC samples. **(A)** Promoter methylation of TFF3 in 450K data from TCGA (cg21970261, CpG site #4) in AN (n=50) vs. PC (n=497) samples; **(B)** Promoter methylation of TFF3 in 450K data from TCGA (cg14283447) in AN (n=50) vs. PC (n=497) samples; **(C)** ROC curve analysis of cancer specificity of TFF3 promoter methylation in TCGA AN vs. PC samples for Illumina CpG site cg21970261; **(D)** ROC curve analysis of cancer specificity of TFF3 promoter methylation in TCGA AN vs. PC samples for Illumina CpG site cg14283447. AN, adjacent normal. PC, prostate cancer. P, p-value (Mann Whitney U-test). Grey line: median methylation.

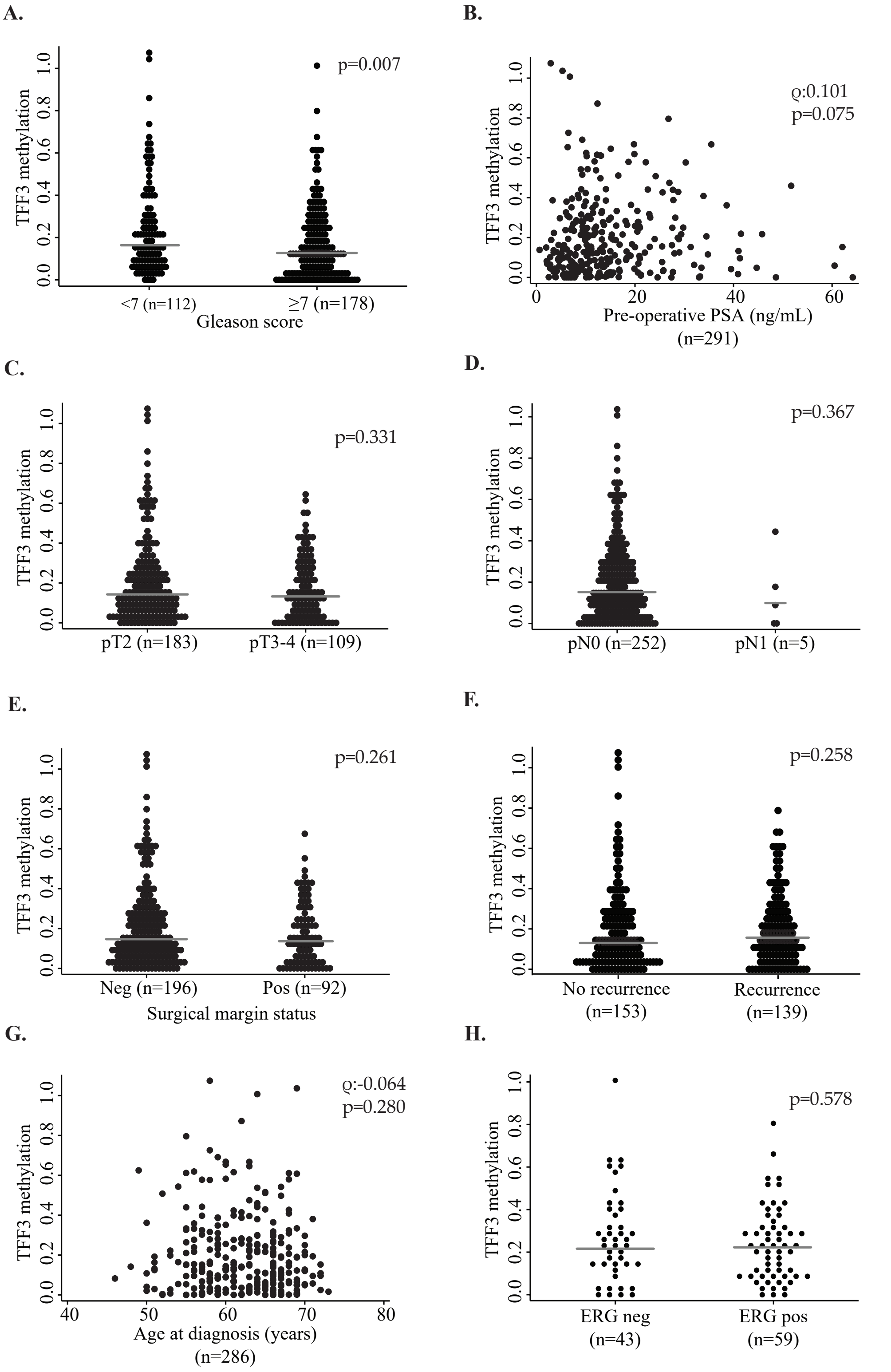

Figure S2: Correlation between *TFF3* methylation and clinicopathological variables in the RP cohort. Correlation between *TFF3* methylation (CpG sites #6-8) and **(A)** Gleason score (n=290); **(B)** Pre-operative serum PSA level (n=291); **(C)** Pathological T-stage (n=292); **(D)** pathological lymphnode stage (n=257); **(E)** Surgical margin status (n=288); **(F)** PSA recurrence status (n=192); **(G)** Age at diagnosis (n=286); **(H)** ERG status (n=102). ϱ, spearman's rho. P, p-value (Mann Whitney U-test or Spearman correlation test). pT, pathological T-stage. pN, pathological lymphnode stage.

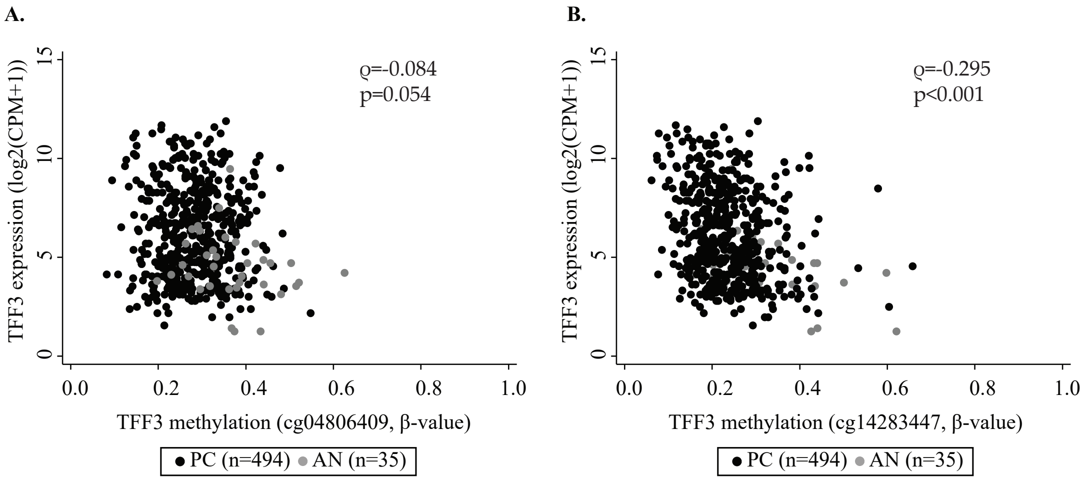

Figure S3: Correlation between *TFF3* promoter hypomethylation and RNA expression in 35 AN and 494 PC samples from TCGA. **(A)** Correlation between TFF3 promoter methylation (cg04806409) and TFF3 RNA expression. (**B)** Correlation between TFF3 promoter methylation (cg14283447) and TFF3 RNA expression. AN, adjacent normal; PC, prostate cancer. P, p-value (spearman's correlation). ϱ, spearman's rho.

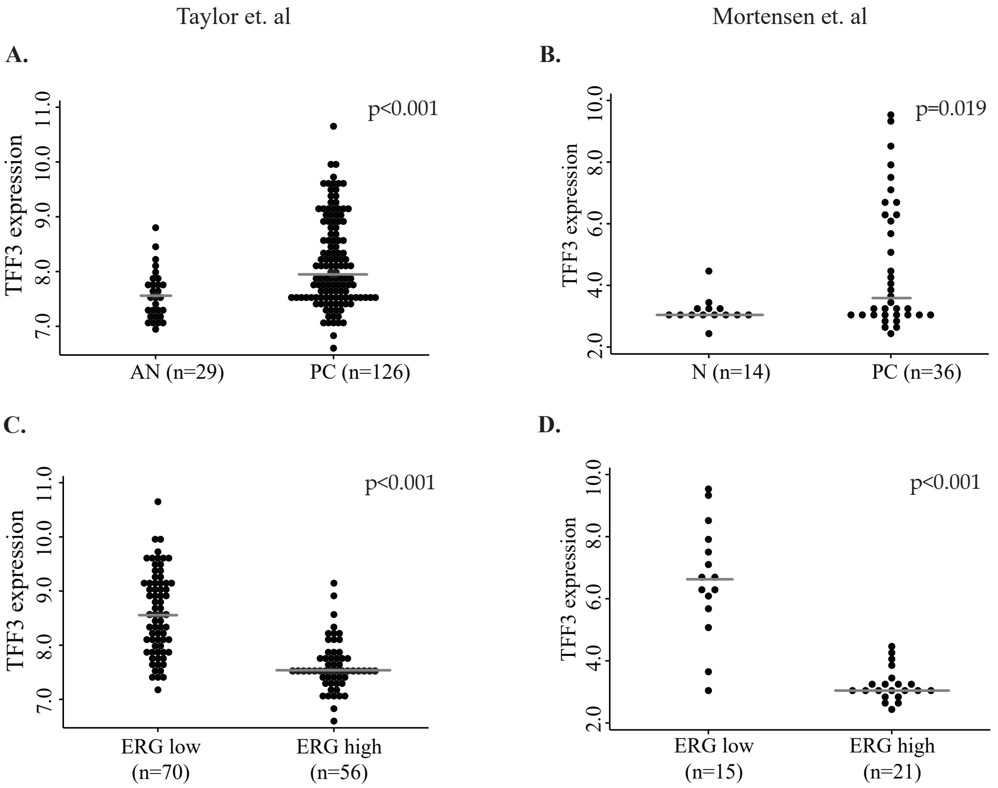

Figure S4: TFF3 RNA expression and correlation to clinicopathological parameters in public microarray data. **(A)** RNA expression of TFF3 in AN (n=29) and PC (n=126) samples from Taylor et al.; **(B)** RNA expression of TFF3 in N (n=14) and PC (n=36) samples from Mortensen et al. Correlation between TFF3 RNA expression and **(C and D)** ERG RNA expression (n=126 and 36, respectively). AN, adjacent normal. PC, prostate cancer. N, normal. P, p-value (Mann Whithney U-test). Grey line: median expression.

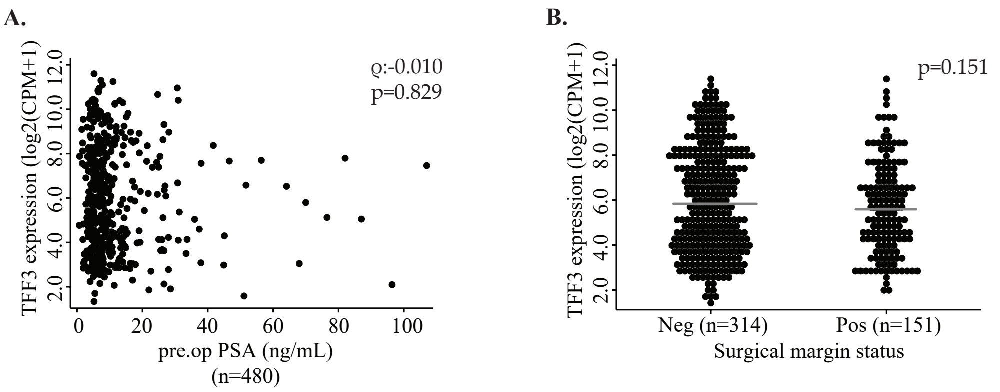

Figure S5: Correlation between TFF3 expression and clinicopathological parameters in RNAseq data from TCGA. Correlation of TFF3 RNA expression and **(A)** Preoperative PSA (n=480); **(B)** Surgical margin status (n=465). CPM, counts per million. P, p-value (Spearman correlation test or Mann Whitney U-test). ϱ, spearman's rho. Pre-op, Preoperative PSA. Grey line: median expression.

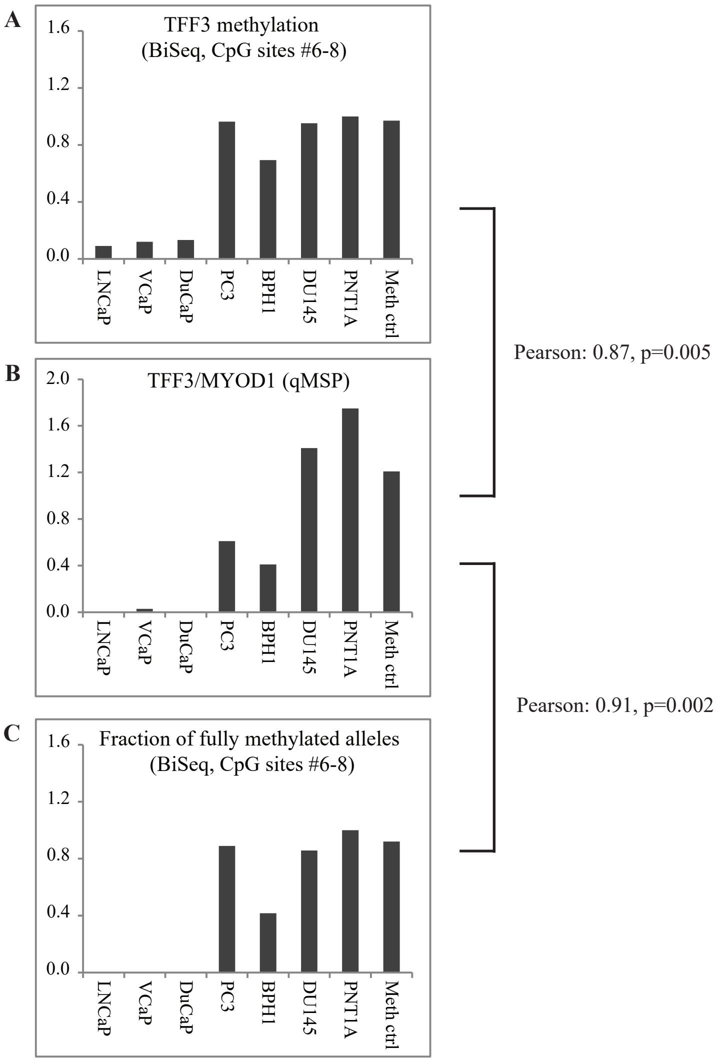

Figure S6: Correlation between *TFF3* methylation quantified by qMSP and bisulfite sequencing analyses, respectively in 7 prostate (cancer) cell lines. **(A)** Mean methylation level for 3 CpG sites (CpG sites #6-8, Figure 2A) in 7 prostate cell lines calculated from previously published BiSeq data (Vestergaard, E.M., et al., Int J Cancer, 2010). Pearson's correlation coefficient to qMSP results in B is given; **(B)** qMSP results for 7 prostate cell lines; **(C)** Fraction of fully methylated alleles for 3 CpG sites (CpG sites #6-8, Figure 2A) calculated from previously published BiSeq data (Vestergaard, E.M., et al., Int J Cancer, 2010). Pearson's correlation to qMSP results in B is given. BiSeq, bisulfite sequencing P, p-value (Pearsons correlation test).
